# Environmental enrichment during forced abstinence from cocaine self-administration opposes gene network expression changes associated with the incubation effect

**DOI:** 10.1038/s41598-020-67966-8

**Published:** 2020-07-09

**Authors:** Gregory L. Powell, Annika Vannan, Ryan M. Bastle, Melissa A. Wilson, Michela Dell’Orco, Nora I. Perrone-Bizzozero, Janet L. Neisewander

**Affiliations:** 1 School of Life Sciences, Arizona State University, PO Box 874501, Tempe, AZ 85287-4501 USA; 2 Center for Evolution and Medicine, Arizona State University, Tempe, AZ USA; 3 Department of Neurosciences, University of New Mexico School of Medicine, Albuquerque, NM USA; 4 Present Address: Nash Family Department of Neuroscience, Icahn School of Medicine at Mount Sinai, New York, NY USA

**Keywords:** Reward, Operant learning, Addiction

## Abstract

Environmental enrichment (EE) is a robust intervention for reducing cocaine-seeking behaviors in animals when given during forced abstinence. However, the mechanisms that underlie these effects are not well-established. We investigated the adult male rat transcriptome using RNA-sequencing (RNA-seq) following differential housing during forced abstinence from cocaine self-administration for either 1 or 21 days. Enriched, 21-day forced abstinence rats displayed a significant reduction in cocaine-seeking behavior compared to rats housed in isolation. RNA-seq of the nucleus accumbens shell revealed hundreds of differentially regulated transcripts between rats of different forced abstinence length and housing environment, as well as within specific contrasts such as enrichment (isolated 21 days vs. enriched 21 days) or incubation (isolated 1 day vs. isolated 21 days). Ingenuity Pathway Analysis affirmed several pathways as differentially enriched based on housing condition and forced abstinence length including *RELN*, the *Eif2* signaling pathway, synaptogenesis and neurogenesis pathways. Numerous pathways showed upregulation with incubation, but downregulation with EE, suggesting that EE may prevent or reverse changes in gene expression associated with protracted forced abstinence. The findings reveal novel candidate mechanisms involved in the protective effects of EE against cocaine seeking, which may inform efforts to develop pharmacological and gene therapies for treating cocaine use disorders. Furthermore, the finding that EE opposes multiple pathway changes associated with incubation of cocaine seeking strongly supports EE as a therapeutic intervention and suggests EE is capable of preventing or reversing the widespread dysregulation of signaling pathways that occurs during cocaine forced abstinence.

## Introduction

Cocaine use disorders (CUDs) are a persistent, costly epidemic within the United States as evidenced by increased numbers of new cocaine users and cocaine-related overdose in the latter half of the 2010s^1^. Attempts to curtail illicit drug use with public policies^2^, law enforcement^3^ and incarceration^4^ have had little success at reducing CUDs. Meanwhile, extensive preclinical^5^ and clinical^6^ research toward developing pharmacological treatments has also had limited success. Unfortunately, with the onset of the opioid crisis in the United States, there has been a parallel increase in cocaine-related deaths^7^, highlighting the need to find novel targets for treatment development.

Insight into novel targets may be gained from examining transcriptomic changes in rodent models that manipulate the degree of motivation for drugs. For instance, motivation for drug is affected by length of forced abstinence. In humans, after a cocaine binge there is initially little if any craving for drug; however, craving emerges within hours to days of abstinence and is often triggered by exposure to drug-associated cues^7^. In parallel, we found that cocaine-seeking behavior elicited by drug-associated cues in rats becomes increasingly stronger over 3–4 weeks of forced abstinence^8,9^. Grimm et al.^11^ coined the term “incubation” to describe this effect and found that it persists for up to 2 months of forced abstinence^10^. Mechanisms contributing to the incubation effect include plasticity within brain regions involved in reward learning and memory, which play a critical role in the development of CUDs^8,9, 11–15^.

Environmental enrichment (EE) is another robust manipulation for reducing motivation to seek and take drugs in rodents as demonstrated for psychostimulants^16–19^, heroin^20^, and nicotine^21–26^. EE typically involves housing animals with social companions along with the opportunity for exercise and exploration of novel items. At the opposite end of the spectrum of housing conditions is isolation, which typically enhances motivation for drug. Compared to standard group housing for instance, isolation enhances cocaine self-administration^27^, whereas EE reduces cocaine-induced hyperactivity^28–30^. Housing conditions contribute to individual differences in cocaine self-administration, exemplified by Puhl et al.,^31^ who found that 96% of rats living in EE qualified as “low drug-takers” compared to 75% of rats living in isolation. Furthermore, EE given as an intervention during forced abstinence from cocaine self-administration reduces cue reinstatement of operant cocaine-seeking behavior compared to both group and isolation housing^32,33^.

Previous research using RNA-seq to examine transcriptomic changes related to the protective effects of EE on reducing cocaine intake in rats during self-administration identified novel pathways that had not been previously implicated in the drug abuse literature^34,35^, including the retinoic acid pathway. However, transcriptomic changes related to the protective effects of EE as an intervention during forced abstinence to reduce motivation to seek cocaine have not been investigated. The present study aimed to address this question by using RNA-seq in the nucleus accumbens shell (NAcsh) of male rats to examine transcriptomic differences associated with varying degrees of cocaine-seeking behavior. We focused on the NAcsh because this striatal region is critically involved with motivation to seek cocaine^36,37^ and shows correlations between changes in addiction-related genes and cocaine-seeking behavior^38–40^. We manipulated motivation levels utilizing isolation and EE housing conditions during forced abstinence for either 1 or 21 days. We expected variation in cocaine-seeking behavior across groups during a test for cue reactivity, with 21 days of forced abstinence in isolation resulting in robust cocaine seeking and brief forced abstinence in EE resulting in the least cocaine seeking.

## Results

### Self-administration and cue reactivity

**Figure 1:**
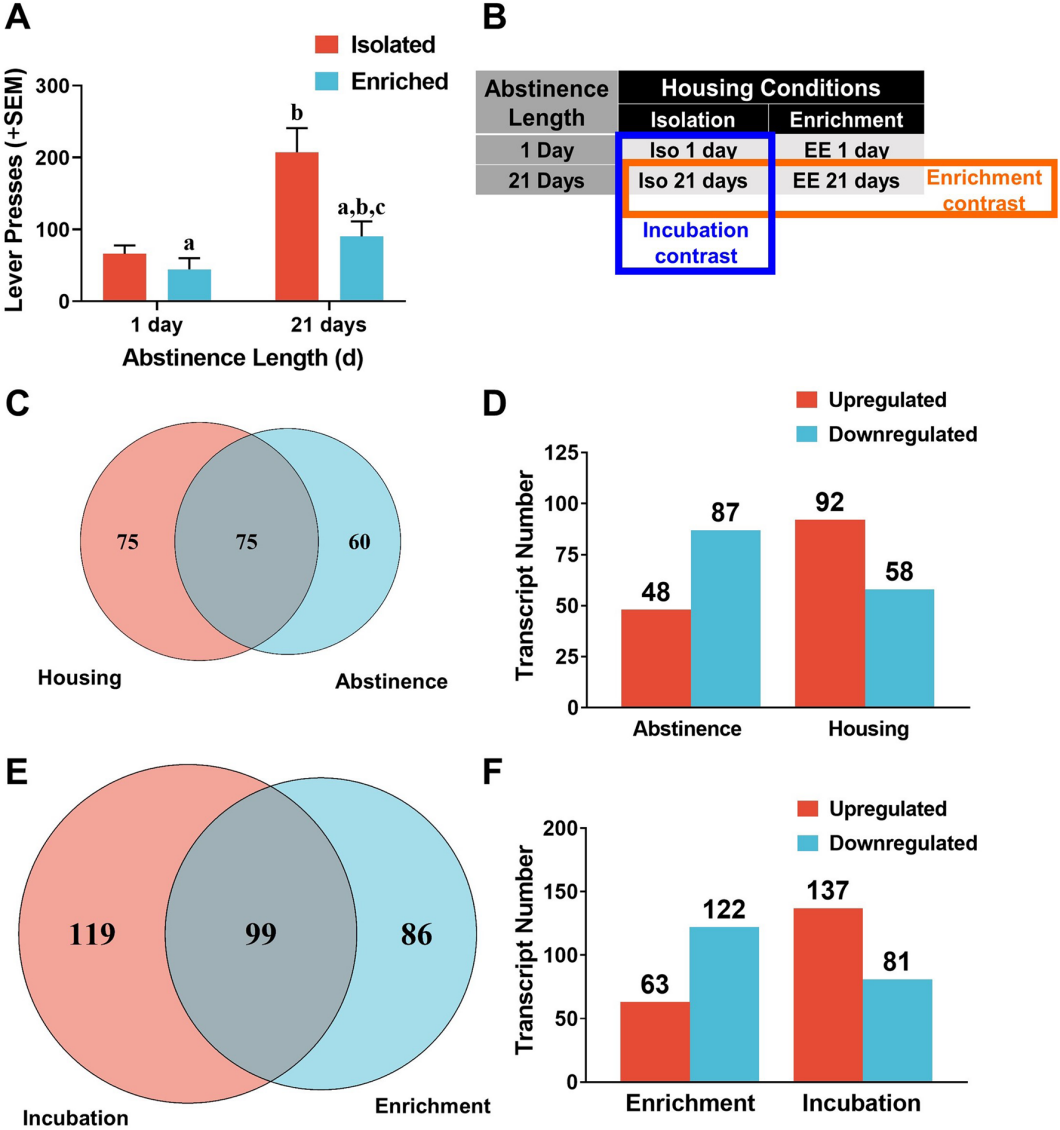
(**A**) Cocaine-seeking behavior shown as the number of lever presses (+ SEM) on the cocaine-associated, active lever during cue reactivity testing following abstinence. Isolated animals pressed the active lever more than enriched animals (*a,* main effect of housing); animals abstinent for 21 days pressed the active lever more than animals abstinent for 1 day (*b*, main effect of abstinence length); and animals isolated for 21 days pressed more than animals in an enriched environment for 21 days (*c,* Bonferroni *t*-test, *p* < 0.025). (**B**) A table explaining the primary comparisons. (**C**) Of the transcripts differentially expressed due to either main factor (abstinence or housing), 75 were shared between them. (**D**) The number of transcripts differentially expressed between the two main factors, abstinence and housing. (**E**) Among the transcripts differentially expressed between the enrichment and incubation contrasts, 99 transcripts were shared between them. (**F**) The number of transcripts differentially expressed due to enrichment (IC21 vs. EC21) or incubation (IC1 vs. IC21).

Following a minimum of 21, 2-h sessions of cocaine self-administration (0.75 mg/kg, IV per infusion), assignment to housing and forced abstinence length conditions was counterbalanced to equate previous consumption and active lever presses (Figure S1) across groups. Rats either remained in isolation (IC) or were placed into EE for either 1 (1D) or 21 days (21D) of forced abstinence (n = 9/housing condition for 1D; n = 15–16/housing condition for 21D), after which all rats underwent a 1-h cue reactivity test. During the test, light/tone cues previously paired with cocaine delivery were presented response-contingently but cocaine was not available (Fig. 1A). A two-factor ANOVA of the number of active lever presses during the test revealed a main effect of forced abstinence length [F_1,45_ = 12.13, *p* < 0.05] with rats abstinent for 21 days exhibiting more active lever presses than those abstinent for 1 day. In addition, there was a main effect of housing [F_1,45_ = 6.67, *p* < 0.05] with IC rats exhibiting more active lever presses than EE rats. There was no housing × forced abstinence length interaction; however, pairwise comparisons using Bonferroni-corrected t-tests revealed that in animals abstinent for 21 days, cue reactivity was greater when they were housed in isolation (IC21D) than when housed in enrichment (EE21D) [t_30_ = 3.60, *p* < 0.0167], whereas there was no difference between housing conditions in animals abstinent for only 1 day (IC1D vs. EE1D).

### RNA-Seq

Following the 1-h cue reactivity test, bilateral micropunches of the NAcsh were pooled from each animal from a subset of animals [N = 12 assigned to one of 4 groups: IC1D, IC21D, EE1D, and EE21D (n = 3/group)] and analyzed for changes in transcript expression. An average of 52 (± 3.3 SEM) million reads were mapped per sample. Of the 41,064 transcripts in the ENSEMBL rat database, we consistently detected 27,942 transcripts in our samples using a cutoff of 1 read across all samples. Based on the Wald test statistic, 135 transcripts were differentially regulated between abstinence lengths regardless of housing and 150 transcripts were differentially regulated between the housing conditions during abstinence prior to testing regardless of abstinence length (Fig. 1B). Among the 285 total transcripts differentially regulated by these factors, 75 were shared between housing conditions and forced abstinence length (Fig. 1C, D). Among the transcripts regulated between forced abstinence lengths (1 vs. 21 days ignoring housing; n = 6/condition), more were downregulated than upregulated, whereas the opposite pattern was observed among those regulated between housing conditions (Isolation vs. EE ignoring abstinence length; n = 6/condition). Using the *contrast* function of ‘DESeq2′ in R, the number of differentially expressed transcripts in the isolation vs. enrichment groups following 21 days of forced abstinence were quantified; this contrast focused on enrichment as an intervention (Fig. 1B, Enrichment contrast; IC21D vs. EE21D; n = 3/group). We also quantified the number of differentially expressed transcripts in the 1 vs. 21 days of forced abstinence groups that remained isolated; this contrast focused on the incubation of motivation for cocaine across time during abstinence (Fig. 1B, Incubation contrast; IC1D vs. IC21D; n = 3/group). 185 transcripts were differentially regulated in the Enrichment contrast and 218 were differentially regulated in the Incubation contrast. Among these 403 differentially-expressed (DE) transcripts, 99 were shared among Enrichment and Incubation (Fig. 1E). Among the transcripts differentially regulated within the Enrichment contrast, more were downregulated than upregulated, whereas the opposite pattern was observed among those regulated within the Incubation contrast (Fig. 1F).

### Forced abstinence effect

**Figure 2:**
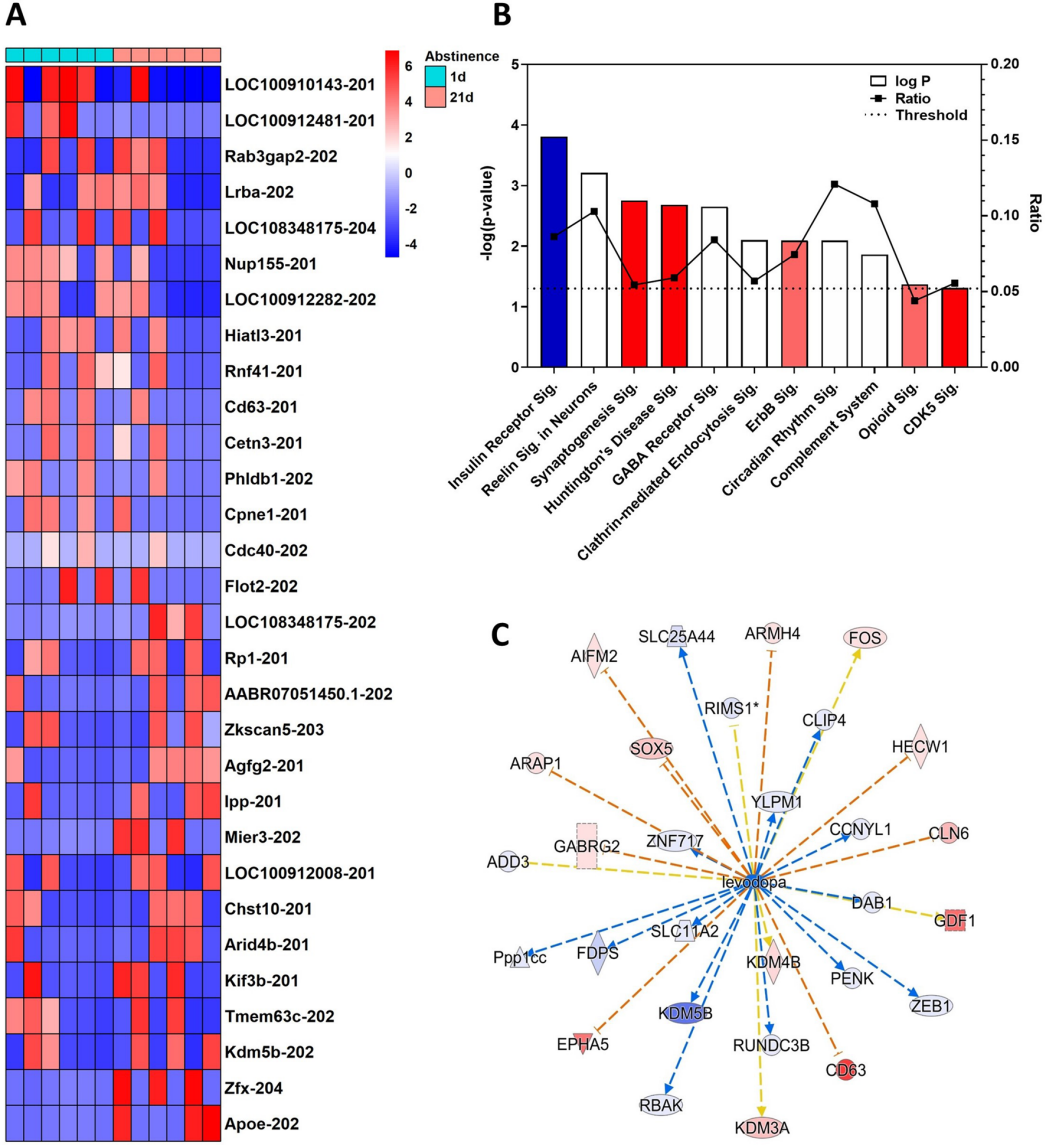
Abstinence-regulated transcripts. (**A**) The top 30 up- (red) and down-regulated (blue) transcripts due to abstinence, according to log2 fold change with subjects abstinent for 1 day denoted by turquoise squares and those abstinent for 21 days by salmon squares. (**B**) A selection of top canonical pathways found using IPA analysis. Blue = negative z-score and impact on the pathway; red = positive z-score and impact on the pathway; white = z-score = 0 signifying no information on impact or no change on the pathway. Log P: the negative logarithm of the *p* value for each pathway. An alpha (P) of 0.05 is equal to 1.301. Ratio: percentage of transcripts in the pathway. Threshold: pathways with a −log(P) > 1.301 were considered significantly regulated. Sig.: Signaling Pathway (**C**) A top upstream regulator according to IPA, *levodopa*, and the transcripts it targets within the Abstinence effect. The color intensity of the symbols represents the fold change of up- (red) or downregulation (blue) of each gene by abstinence.

Of the 135 transcripts regulated within the forced abstinence contrast (1D vs. 21D), the top 30 up- or down-regulated transcripts by log2 fold change can be seen in Fig. 2A. An additional approach to examining the forced abstinence contrast was to use Ingenuity Pathway Analysis (IPA) to investigate transcripts with *p* ≤ 0.05 from the DESeq2 analysis described above and the effect of changes in these transcripts in pathway activation or inhibition is shown as positive or negative Z scores, respectively. A total of 693 transcripts met this criterion for the “Forced Abstinence” effect; following removal of duplicate genes by IPA a total of 586 transcripts remained. Selected pathways differentially regulated in forced abstinence are shown in Fig. 2B, with the “threshold” representing the negative log of the alpha-level (0.05) and “ratio” representing the number of differentially expressed transcripts in each canonical pathway vs. total number of transcripts in each category (i.e., a ratio of 0.10 is equivalent to 10% genes in the pathway). The top-ranked canonical pathways included *Insulin Receptor Signaling, Reelin Signaling in Neurons,* and *Synaptogenesis Signaling in Neurons*. All output data from IPA analysis of the abstinence effect can be found in the Table S1.

### Synaptogenesis signaling in neurons

One significant canonical pathway within the forced abstinence effect, *Synaptogenesis Signaling in Neurons,* was of particular interest. The differentially regulated molecules within the pathway that are driving this effect (activation *Z-*score: 1.213) are predicted to be spread across the synaptic cleft. This activation Z-score indicates that the pathway is predicted to have increased activity in animals that experienced prolonged forced abstinence. In the presynaptic bouton, calcium/calmodulin-dependent protein kinase II gamma (*Camk2g*), adenylate cyclase 6 (*Adcy6*), synaptotagmin 17 (*Syt17*), adaptor related protein complex 1 subunit beta 1 (*Ap1b1*), and heat shock protein family A (*Hsp70*) member 8 (*Hspa8*) are all upregulated (Table S1). At the postsynaptic membrane, cadherin 13 (*Cdh13*) and neuroligin 2 (*Nlgn2*), both involved with scaffolding of the postsynaptic region, are downregulated. Additionally, the mammalian target of rapamycin kinase mTor (*Mtor*) is upregulated in abstinence, is part of the *Synaptogenesis Signaling in Neurons* pathway, and is known to influence synaptic spine development and maturation as well as local protein translation in dendrites^41,42^.

### Other functions and pathways related to forced abstinence

In addition to canonical pathways, IPA predicts upstream regulators of the molecules and expression levels that are input. Among the top upstream regulators, the dopamine precursor levodopa (L-dopa) was predicted to be in an inhibited state (Z-score = − 3.023, *p* = 3.16E−03; Fig. 2C). Additionally, *Reelin signaling in neurons* (*p* = 6.15E−04, ratio = 0.103) was among the top-ranked canonical pathways regulated by forced abstinence length, although reelin (*Reln*) itself was unchanged due to forced abstinence. Lastly, Figure S2 illustrates a selection of the top Diseases and Functions categories enriched within the forced abstinence effect. Within the regulated *Behavior* category, subcategories such as *Cognition* (*p* = 4.51e−6, z-score: −0.074), *Learning* (*p* = 1.39e−6, z-score: − 0.242), and *Spatial learning* (*p* = 1.21e−7, z-score: −0.923) are all predicted to have been negatively impacted by prolonged abstinence duration.

### Housing effect

**Figure 3:**
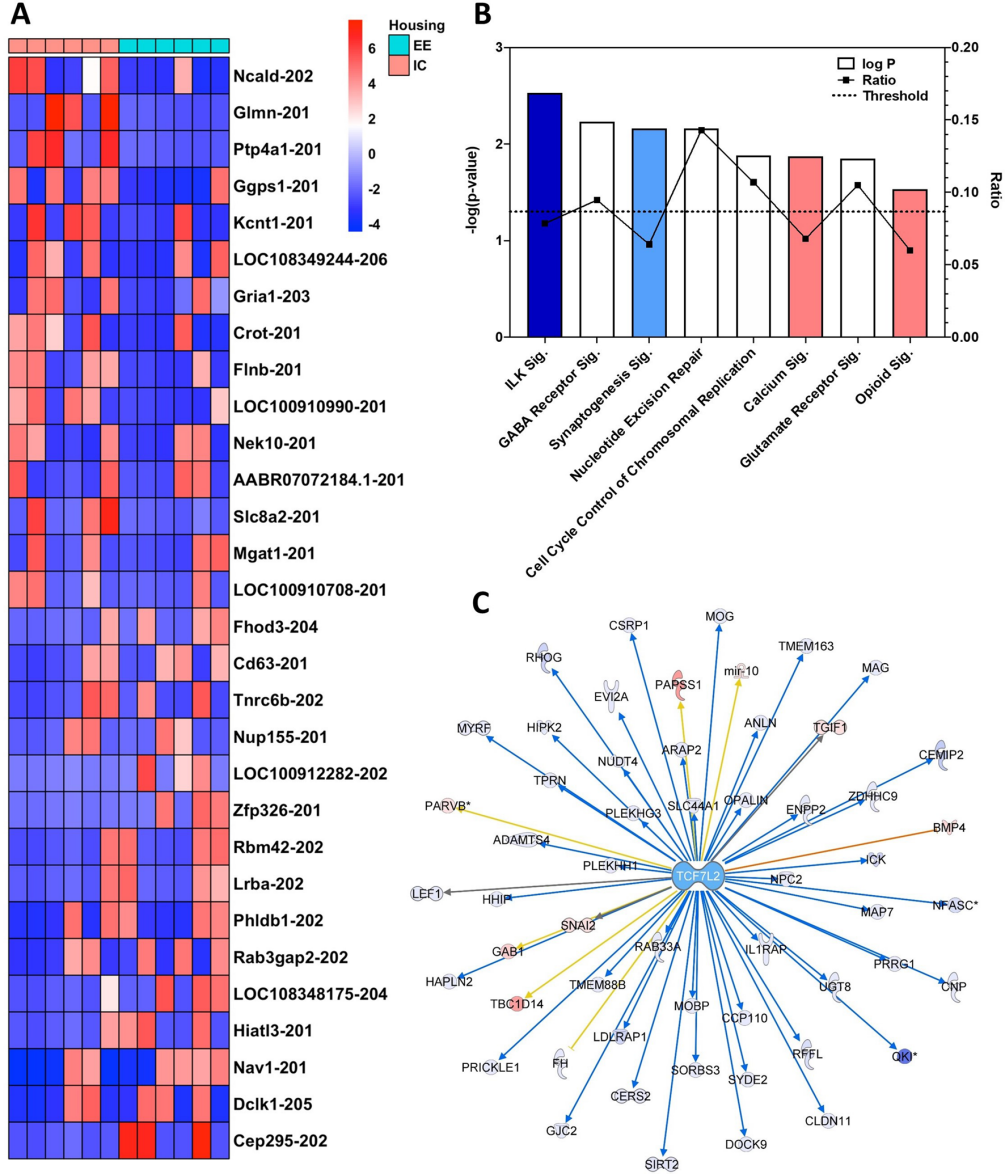
Housing-regulated transcripts. (**A**) The top 30 up- and down-regulated transcripts due to housing, with subjects in enriched housing denoted by turquoise squares and those in isolation housing by salmon squares. (**B**) A selection of top canonical pathways found using IPA analysis. Blue = negative z-score; red = positive z-score; white = z-score = 0. Ratio: percentage of transcripts in the pathway targeted. Sig.: Signaling Pathway (**C**) TCF7L2, a transcriptional regulator that targets 55 molecules found in the housing effect, was the top upstream regulator. The color intensity of the symbols represents the fold change of up- (red) or downregulation (blue) of each gene by housing environment.

From the 150 differentially expressed transcripts due to the housing main effect (IC vs. EE), the top 30 up- or down-regulated transcripts are shown in Fig. 3A. Using the 847 transcripts with *p* ≤ 0.05 from the DESeq2 analysis, the top-ranked canonical pathways of interest for the housing main effect were *Integrin-Linked Kinase (ILK) Signaling* (Fig. 3B) (*p* = 2.96E−03, *Z*-score: −1.069), *GABA Receptor Signaling* (*p* = 5.94E−03; no activity pattern available), and *Synaptogenesis Signaling Pathway* (*p* = 6.91E−03; *Z*-score: −0.229). All output data from IPA analysis of the housing effect can be found in the Table S2.

### ILK signaling

The top canonical pathway, *ILK Signaling*, has a predicted inhibition based on transcript expression results from our RNA-seq analysis. The molecules involved represent approximately 7.9% of the pathway and include integrin subunit beta 4 (*Itgb4*), *Mtor*, and several other molecules included in the pathway that recent research has shown to be crucial for the establishment of neuronal polarity^43^.

### Other functions and pathways related to housing

Among the top predicted upstream regulators of the effect of housing, the transcriptional regulator *Tcf7l2* targeted 55 molecules with *p* ≤ 0.05 (Fig. 3C). Within the Diseases and Biological Functions categories (Figure S3), many *Nervous System Development and Function* categories are predicted to be inhibited, including *Migration* (*p* = 1.18E−07, Z-score: − 2.454), *Development* (*p* = 1.17E−08, *Z*-score: − 3.244), *Proliferation* (*p* = 6.80E−07, *Z*-score: − 2.782), *Branching* (*p* = 1.64E−04, *Z*-score: − 2.259), *Growth* (*p* = 5.13E−07, *Z*-score: − 3.442), and others. Furthermore, within the *Cell Death and Survival* pathway a decrease in *Cell viability* (*p* = 6.04E−07, *Z*-score: − 2.482) and *Cell survival* (*p* = 6.96E−07, *Z*-score: − 2.368) and a concurrent increase in *Apoptosis* (*p* = 7.09E−04, *Z*-score: 2.173) are predicted. These pathways are explored in further detail below in the Incubation and Enrichment contrast results.

### Incubation effect

**Figure 4:**
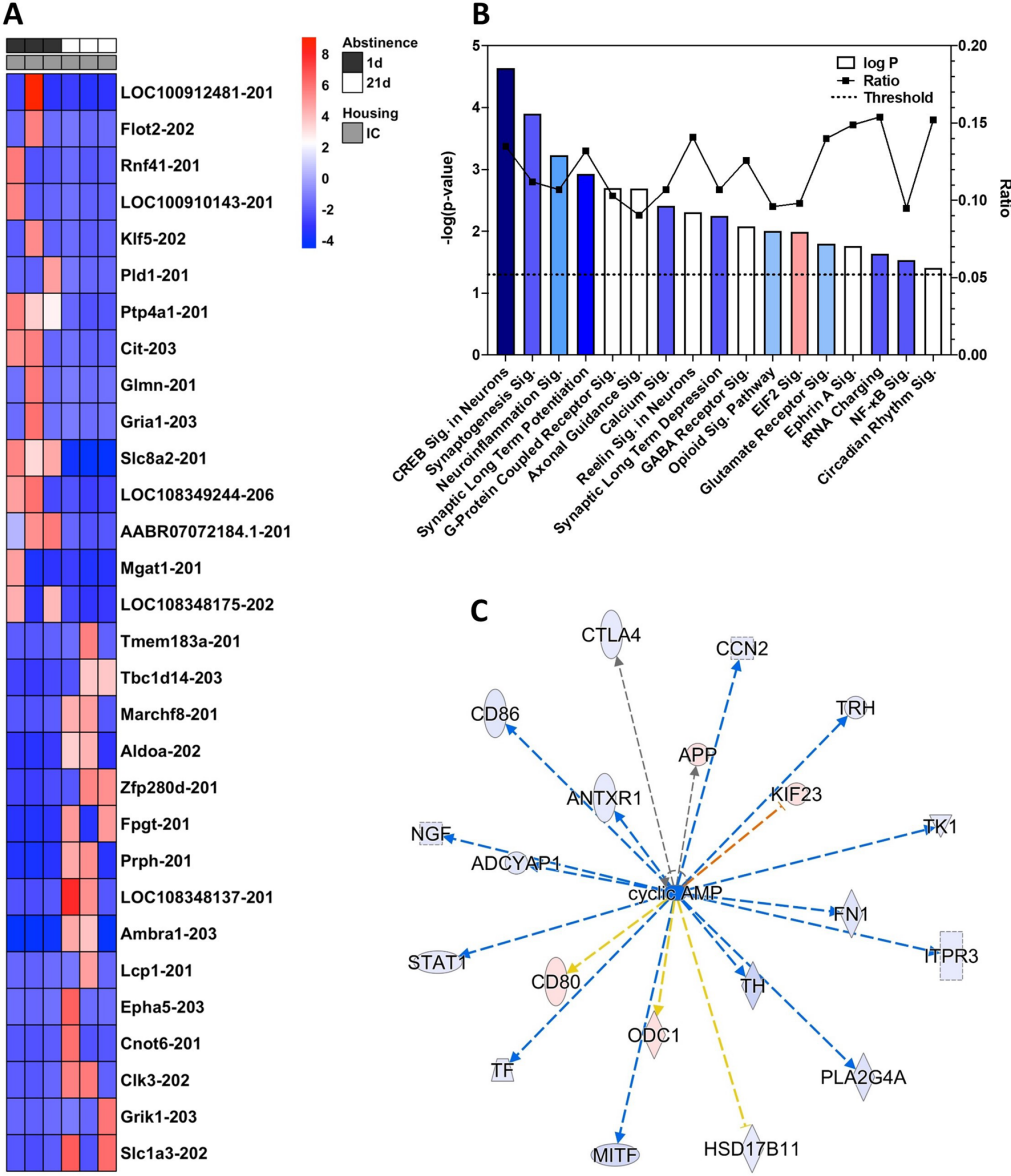
Incubation-regulated transcripts. (**A**) The top 30 up- and down-regulated transcripts due to incubation. (**B**) A selection of top canonical pathways found using IPA analysis. Blue = negative z-score; red = positive z-score; white = z-score = 0. Ratio: percentage of transcripts in the pathway targeted. Sig.: Signaling Pathway (**C**) cAMP was predicted as a top upstream regulator due to its predicted targeting of differentially expressed transcripts. The color intensity of the symbols represents the fold change of up- (red) or downregulation (blue) of each gene by the Incubation effect.

We used the “contrasts” feature of “DESeq2” in R to compare expression data from animals that experienced isolation housing during forced abstinence for 1 or 21 days (IC1D vs. IC21D). The top 30 (of 218 total differentially expressed) up- or down-regulated transcripts can be seen in Fig. 4A. A total of 1,463 transcripts met selection criteria for IPA analysis using a threshold of *p* ≤ 0.05, with 1,403 transcripts remaining after the removal of variants. A selection of the top canonical pathways for the Incubation effect included *CREB Signaling in Neurons* (*p* = 2.27E−05, *Z*-score: − 2.837), *Synaptogenesis Signaling Pathway* (*p* = 1.26E−04, *Z*-score: − 1.061), and *Synaptic Long Term Potentiation* (*p* = 1.17E−03, *Z*-score: − 2.5). All output data from IPA analysis of the incubation effect can be found in the Table S3.

### CREB signaling in neurons

*Cyclic-AMP response element binding protein (CREB) Signaling in Neurons*, the top canonical pathway for the Incubation effect (Fig. 4B), is led by the significant upregulation of *Creb* itself in prolonged abstinence. CREB has long been linked to memory, plasticity, neuronal development and survival, and has gained recent attention for its connection to neurological disorders^44^. Our results indicate that while *Creb* is upregulated in Incubation, other genes in the pathway including *Camk2d/g* and RNA polymerase II subunit A (*Polr2a*) are all downregulated. Combined with the other affected molecules, IPA predicts an inhibition of *CREB Signaling in Neurons*.

### Other functions and pathways related to incubation contrast

In addition to the pathways described above, many other significant pathways showed similar *Z*-scores indicating inhibited action, including *Neuroinflammation Signaling Pathway* (*p* = 5.87E−04; activated *Z*-score: − 0.577), *Calcium Signaling* (*p* = 3.89E−03; *Z*-score: − 1.5), *Synaptic Long Term Depression* (*p* = 5.63E−03; *Z*-score: − 1.886), *Glutamate Receptor Signaling* (*p* = 1.58E−03; *Z*-score: − 0.447), *Opioid Signaling Pathway* (*p* = 9.86E−03; *Z*-score: − 0.853), and *NF-κB Signaling* (*p* = 2.93E−02; *Z*-score: − 1.5). Conversely, the *EIF2 Signaling Pathway* that regulates translation initiation was predicted to be activated by prolonged abstinence (*p* = 1.02E−02; *Z*-score: 0.535). Among the top upstream regulators, cyclic AMP (cAMP) signaling was predicted to regulate 20 transcripts that show differential expression between durations of abstinence in isolation (Fig. 4C), with three quarters of those transcripts downregulated. The upstream analysis also highlighted the importance of transcription regulation within the incubation effect, as 90 of 542 predicted upstream regulators are classified as “transcription regulator” molecules, the most of any category. Using the Diseases and Functions analysis function of IPA, top-regulated categories include *Cellular Assembly and Organization*, *Cellular Function and Maintenance*, *Cell Death and Survival*, *Nervous System Development and Function*, and *Neurological Disease* (Figure S4).

### Enrichment effect

**Figure 5:**
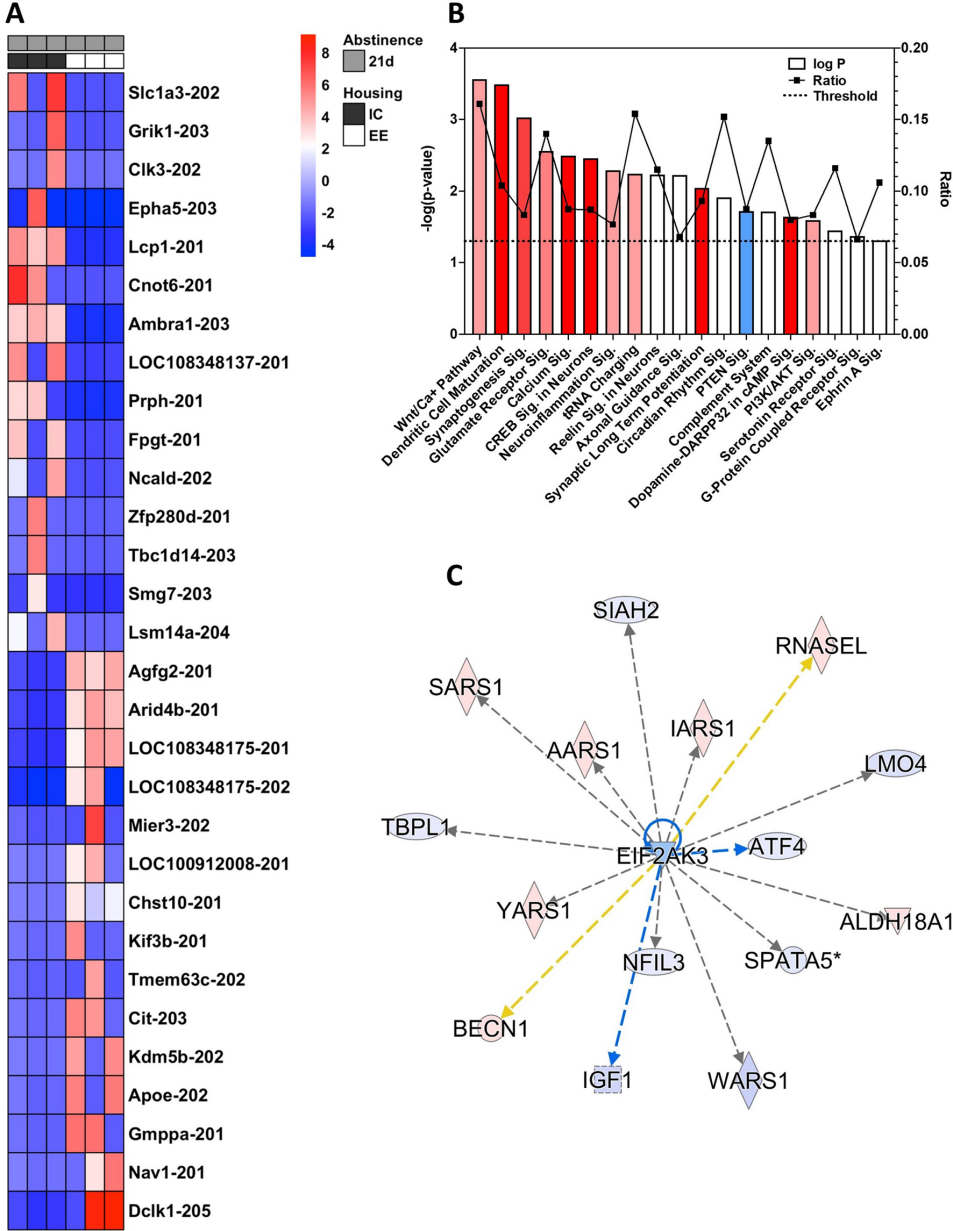
Enrichment-regulated transcripts. (**A**) The top 30 up- and down-regulated transcripts due to enrichment. (**B**) A selection of top canonical pathways found using IPA analysis. Blue = negative z-score; red = positive z-score; white = z-score = 0. Ratio: percentage of transcripts in the pathway targeted. Sig.: Signaling Pathway (**C**) EIF2AK3 was predicted as a top upstream regulator and is known to regulate protein translation. The color intensity of the symbols represents the fold change of up- (red) or downregulation (blue) of each gene by the Enrichment effect.

The top 30 up- or down-regulated transcripts identified from the “enrichment effect” contrast comparing animals in forced abstinence from cocaine for 21 days in either isolation or enriched housing (IC21D vs. EE21D) can be seen in Fig. 5A. We used IPA with the 1,091 transcripts with a *p* ≤ 0.05 from the DESeq2 analysis and further trimmed to 1,040 transcripts to remove duplicate transcript variants. A selection of the top canonical pathways (Fig. 5B) includes *Wnt/Ca*^+^ *Pathway* (*p* = 2.73E−04; *Z*-score: 0.632), *Dendritic Cell Maturation* (*p* = 3.24E−04; *Z*-score: 2.183), and *Synaptogenesis Signaling Pathway* (*p* = 9.38E−04; Z-score: 1.8). All output data from IPA analysis of the enrichment effect can be found in the Table S4.

### Eukaryotic initiation factors

Within the analysis of predicted upstream regulators of the enrichment effect one of the top molecules was *Eif2ak3* (also known as *Pek* or *Perk*), a kinase of the eukaryotic initiation factor 2 alpha (eIF-2-α; Fig. 5C). Within the enrichment data obtained here, activity of *Eif2ak3* is predicted to be inhibited, suggesting phosphorylation of eIF-2-α is down when translation and cocaine-seeking are up. However, normalized expression counts from the enrichment comparison indicate that another eIF-2-α kinase, *Eif2ak2* (also known as *Pkr*, *Ppp1r83*, or *Prkr*), showed increased expression in EE rats, as does *Eif4e2*. Thus, overall phosphorylation of *Eif* signaling is potentially increased in enrichment, which would lead to a reduction in protein translation according to the established mechanisms^45^.

### Other functions and pathways related to the enrichment contrast

In addition to the IPA pathways described above, several other pathways of interest were significantly affected: *Glutamate Receptor Signaling* (*p* = 2.76E−03; *Z*-score: 0.447), *CREB Signaling in Neurons* (*p* = 3.44E−03; *Z*-score: 2.668), *Calcium Signaling* (*p* = 3.27E−03; *Z*-score: 2.309), *Synaptic Long Term Potentiation* (*p* = 9.21E−03; *Z*-score: 2.887), and *Neuroinflammation Signaling Pathway* (*p* = 5.07E−03; *Z*-score: 1.606) (Fig. 5B). Many of these pathways were positively activated with EE compared to isolation, the opposite of that seen with the incubation effect above. Finally, IPA analysis indicates that Diseases and Functions significantly regulated by the Enrichment effect include similar groupings as seen above for the incubation effect, including *Nervous System Development and Function, Neurological Disease, Cellular Assembly and Organization*, *Cellular Function and Maintenance*, and *Cell Death and Survival*, as well as *Post-Translational Modification* and *Protein Synthesis* (Figure S5)*.*

### RT-qPCR results

To validate the effects seen using RNA-seq, a selection of transcripts from a subset of the rats (21 days of forced abstinence; isolation: n = 6, enrichment: n = 7) was screened using RT-qPCR. RNA-seq results for *Camk2d* (ENSRNOT00000016026) showed a 9.01-log2 fold increase that was validated as a 1.30-fold increase using RT-qPCR (Figure S6A). One of the *Eif4e2* transcript isoforms (ENSRNOT00000026646) showed a log2 fold increase of 7.62 in the RNA-seq analysis that was validated as 1.69-fold increase with RT-qPCR (Figure S6B). It should be noted that RT-qPCR primers were designed from transcript-specific exon sequences. For example, the *Eif4e2* transcript chosen (ENSRNOT00000026646) represents 1 of 6 splice variants in the Ensembl *Rattus norvegicus* library.

## Discussion

The present study identified transcriptome differences in male rats with a history of cocaine self-administration that were housed in isolation or in an enriched environment during forced abstinence from cocaine for either 1 or 21 days. As expected from previous research^32,33^, isolation housing during the 21-day forced abstinence period from cocaine self-administration resulted in robust cocaine-seeking behavior during a cue reactivity test, whereas EE housing, which included social, exercise, and novelty components, blunted cocaine-seeking behavior to levels similar to that observed with only 1 day of forced abstinence (Fig. 1A). A lead hypothesis of the mechanisms underlying the robust effects of EE on cocaine-related behaviors is stress innoculation^46^. Based on this theory, EE presents mild stress that fine tunes stress reactivity systems such that EE rats are better able to cope with the stress. In the present study, EE rats may better cope with stress associated with the cue reactivity test than rats housed in isolation, thereby reducing incentive to seek cocaine. Another feature of the present experiment that introduces differential stress across groups is the switch in environments in EE rats, but not isolated rats, during forced abstinence. Previous research from our lab employing a similar experimental design and timeline showed no differences in plasma corticosterone levels between enriched and isolated saline-yoked animals^47^. Furthermore, a decrease in plasma corticosterone levels was observed in enriched rats compared to isolated rats with a history of cocaine self-administration^47^. These previous findings suggest that EE rats adapt rapidly to potential stress effects of the switch from isolation to EE and suggest that the reduction of cocaine seeking in EE rats may involve stress inoculation.

Given that RNA-seq identifies hundreds of regulated transcripts, it is important to validate findings via other measures of RNA or comparison to previous literature. In this study, the *Signaling by Rho Family GTPases* pathway was downregulated by 21 days of forced abstinence, similar to the results of a recent RNA-seq study of cocaine self-administration where this pathway was reported as the top-ranked canonical pathway^34^. Furthermore, this pathway has been shown to be downregulated by experimenter-delivered cocaine administration^48^. Together, these results suggest that the downregulation of the Rho associated pathway is in part due to cocaine history but potentially amplified by protracted, forced abstinence. Additionally, the retinoic acid receptor activation pathway was differentially regulated by cocaine across the two housing conditions, with upregulation in animals housed in enrichment and downregulation in animals housed in isolation. Similarly, Zhang and colleagues^34^ previously found significant upregulation of the retinoic acid pathway due to housing in EE prior to cocaine self-administration. In addition, *Camk2d* and *Eif4e2* transcripts were chosen for validation from a cohort of animals that experienced the same cocaine self-administration protocol and 21 days of abstinence in either isolation or enrichment. Both of these transcripts demonstrated fold increases in accordance with the RNA-seq data (Figure S6). These findings together with the consistency of the present RNA-seq data with the previous literature offers validation of our analytical approach.

An exciting result from the RNA-seq analysis is that the robust transcriptomic differences observed with incubation (i.e., 1 vs. 21 days forced abstinence contrast in isolated rats) showed largely opposite changes with enrichment (i.e., EE versus isolation contrast in 21-day forced abstinent rats) within several pathways. Of the 155 regulated pathways in either the incubation or enrichment contrasts, 66 are shared between the two conditions. Furthermore, 49 of those 66 shared pathways show reverse activity levels between incubation and enrichment. For instance, *Synaptic Long Term Potentiation* has significantly upregulated activity in enrichment but downregulated activity in incubation. Additionally, *Synaptogenesis Signaling Pathway* has the identical pattern—upregulated activity in enrichment and downregulated in incubation. Additional pathways that exhibit opposing regulation by incubation versus EE include *CREB Signaling in Neurons*, *Neuroinflammation Signaling Pathway*, *Calcium Signaling*, and *Glutamate Receptor Signaling*, which have been implicated in cocaine-seeking behavior previously^49–53^. The *Opioid Signaling Pathway* was also differentially regulated in the incubation contrast, which is a pathway that has received relatively little attention as a mechanism of cocaine-seeking behavior but includes molecules such as *Camk2d*, *Camk2g*, *Grin2d*, *Th*, and *Nfkb1* as contributing toward its significant regulation. The enrichment contrast implicated the *Wnt/Ca*^++^ *Pathway*, *Dendritic Cell Maturation*, *PI3K/AKT Signaling*, and *Dopamine-DARPP32 Feedback in cAMP Signaling* pathways in the blunting effect of EE on cocaine-seeking behavior. Thus, environmental enrichment may prevent or reverse multiple gene network changes that take place during abstinence and that presumably contribute to incentive motivation for cocaine, which manifests as cocaine craving in individuals with CUD and is thought to be a major factor in relapse.

The IPA analysis suggested additional pathways that exhibit differential regulation across abstinence period regardless of housing conditions and across housing conditions regardless of forced abstinence length. The abstinence effects were observed for *Huntington’s Disease Signaling* and *Reelin Signaling in Neurons* pathways. The reelin pathway is a key contributor to the development of schizophrenia and other neuropsychiatric disorders such as depression and psychosis^54,55^, and these psychiatric conditions are often comorbid with substance use disorders^56,57^. Additionally, the reelin pathway modulates gamma-aminobutyric acid (GABA) transmission and neuroplasticity, two mechanisms thought to be involved in cocaine use disorders^58,59^. Housing condition effects were observed for *ILK Signaling*, a pathway known to be linked to cocaine sensitization and structural plasticity^60^, and *Tcf7l2*, a transcription factor commonly associated with diabetes but recently linked to nicotine consumption^61^.

The top pathways exhibiting whole transcriptome changes related to Diseases and Biological Functions highly overlapped between the incubation and EE contrasts, although the changes were in opposite directions. These include nervous system development, neurological disease, and several cellular functions, such as *neuronal cell death* (increased activity) and *proliferation of neuronal cells* (increased activity) in the enrichment contrast. By contrast, in the incubation contrast (Isolated 1 day vs. Isolated 21 days), the *proliferation of neuronal cells* pathway shows significantly reduced activity with prolonged withdrawal as well as a reduction in the *neuronal cell death* category. Under some experimental manipulations, the ventral striatum acquires new neurons (see review by Gould, 2007). For example, chronic infusion of the dopamine D3 receptor agonist, 7-OH-DPAT, into the subventricular zone leads to increased proliferation of new cells within the striatum of adult female rats^63^. Furthermore, intracerebroventricular delivery of platelet-derived growth factors and brain-derived neurotrophic factor (BDNF) in adult female rats leads to an increase in the number of newly generated neurons within the striatum^64^. While little attention has been given to adult neurogenesis within the nucleus accumbens with drugs of abuse, considerable research has shown that cocaine exposure reduces neurogenesis in the adult hippocampus, including in self-administration studies^65,66^. Interestingly, hippocampal neurogenesis functions have previously been implicated in post-mortem tissues of human cocaine users, with 3 molecules—liprin beta 1 (PPFIBP1), oligophrenin 1 (OPHN1), axotrophin (AXOT)—upregulated by cocaine, and 2 molecules—SH3 and multiple ankyrin repeat domains 2 (SHANK2) and semaphorin 6A (SEMA6A)—downregulated by cocaine^67^. In contrast, EE increases neurogenesis in the dentate gyrus of adult rats as well as a corresponding improvement in spatial memory in a water maze testing paradigm^68^. These findings allude to a loss of plasticity within NAcsh circuits involved in incubation, resulting in enhanced expression of the prepotent drug seeking response, and a “reboot” of plasticity by enrichment, which weakens the prepotency of drug seeking behavior.

An interesting overlap between the present study and previous research examining EE effects on cocaine self-administration is the upregulation of transcripts in the eukaryotic initiation factor (EIF) signaling pathway^34^. Three EIF transcript variants were differentially expressed among our conditions: (1) *Eif1b-201* is increased in prolonged forced abstinence; (2) *Eif4e2-201* is increased in EE; and, (3) *Eif2b2-201* is increased in incubation. *Eif2 signaling* pathway genes were also upregulated in isolated animals abstinent from cocaine for 21 days compared to 1 day, suggesting increased pathway activation. Zhang et al. (2016) previously observed that the top canonical pathway altered by EE housing prior to cocaine access was *Eif2 signaling,* which regulates mRNA translation. Recent studies in mice indicate that when expression levels of phosphorylated eIF-2-α, the protein product of *Eif2s1*, in the ventral tegmental area are reduced through mutation or blocked pharmacologically, vulnerability to nicotine^69^ and cocaine^70^ increases. Furthermore, when tobacco smokers express a single nucleotide polymorphism in the orthologous gene that encodes eIF-2-α in humans (which shares > 99% of the sequence with mice and rats), a reduction in reward-induced activity within the caudate and putamen nuclei is observed^69^. Control of translation by eIF-2-α phosphorylation is critical for regulation of synaptic strength and memory (see review by Costa-Mattioli et al.^71^). These same processes in the amygdala have been shown to mediate reconsolidation of drug-conditioned place preference memory for morphine and cocaine as well as for heroin-associated cue memories following establishment of self-administration^72^. Furthermore, Werner et al. (2018) found that eIF-2-α is dephosphorylated when rats experience a test for cocaine-seeking following withdrawal from cocaine and thus translation is increased; however, when they then blocked dephosphorylation of eIF-2-α to restore inhibition of translation, cocaine seeking was reduced^73^. These findings combined with the effects observed on the *Eif2 signaling* pathway here highlight the importance of cocaine on translational control within the nucleus accumbens and that EE influences those mechanisms in a restorative manner.

Surprisingly, none of our contrasts included a significant portion of the *ERK/MAPK Signaling* pathway even though there are converging lines of evidence that this pathway is involved in cocaine-seeking behavior (see reviews ^74–76^). There are several differences between the previous studies and the present study that may account for this discrepancy, including differences in the location of brain sample^47^, measures of protein vs. transcript^35,77–79^, and differences in experimental design regarding the control comparison (saline control or other cocaine-experienced groups)^34,35^ and when EE was utilized (i.e., prior to self-administration or during abstinence)^34,35,77^. Other studies of ERK changes with drugs of abuse have focused on the amygdala or hippocampus, two regions known to be crucial for the development and persistence of CUD but not tested here (see reviews ^80,81^). Additional investigation in these other brain regions of the complex interaction between housing environment and duration of forced abstinence could potentially reveal region-specific differences in the *ERK/MAPK Signaling* pathway crucial to our understanding of CUD.

In conclusion, EE provided as an intervention during forced abstinence from cocaine self-administration leads to numerous changes in mRNA expression in the NAcsh that impact multiple signaling pathways and neuronal functions, including synaptic and neuronal plasticity as well as neuroinflammation. Additionally, the EE intervention regulates the reelin pathway, similar to previous studies using EE prior to cocaine exposure. Importantly, the present findings suggest two novel pathways—translational control and neurogenesis—within the striatum that are ripe for further exploration using established techniques in drug addiction research. For example, neurogenesis and similar annotations within the *Cellular Growth and Proliferation* category are downregulated in animals with increased motivation, suggesting that treatments that promote neurogenesis given during forced abstinence may help prevent reinstatement. Additionally, the work discussed here illustrates that environmental enrichment reverses multiple dysregulated pathways that underlie drug seeking behavior, particularly the pathways that switched directions of expression between the incubation (isolated 1 day vs. isolated 21 days) and enrichment (isolated 21 days vs. enriched 21 days) groups. Together, these findings suggest that therapies, either behavioral, genetic, or pharmacologic, that target these pathways may be useful treatments for CUDs.

## Methods

### Animals

62 male Sprague–Dawley rats (225–250 g on arrival; Charles River Laboratories, Wilmington, Massachusetts USA) were separated into standard, individual housing upon arrival (21.6 × 45.7 × 17.8 cm) with ad libitum access to food and water. Animals were handled daily for 5 days prior to surgery. All procedures were approved by the Institutional Animal Care and Use Committee of Arizona State University and were in accordance with institutional and NIH guidelines.

### Surgery

Jugular vein catheters were implanted in all rats as described previously^82^ under 2–3% isoflurane anesthesia. Rats were given meloxicam (1 mg/kg/ml, SC) and buprenorphine (0.05 mg/kg/ml, SC) analgesics, and cefazolin (30 mg/kg/ml, SC) antibiotic. The free end of the catheter was tunneled under the skin and cemented to the skull using dental acrylic. Post-operational care continued for a minimum of 5 days and included handling and administration of cefazolin (100 mg/kg/ml, IV).

### Self-administration

Animals initially were food restricted to 16 g standard chow per day with ad libitum access to water. Rats (n = 62) received 2-h cocaine (0.75 mg/kg/0.1 ml, IV) self-administration sessions 6 days per week at the same time of day. Upon completion of operant schedule on the active lever, the light and tone stimuli were activated followed 1-s later by infusion of cocaine administered over 6-s. An inactive lever was also available and presses were recorded but produced no consequences. A 20-s timeout with a lit house light followed termination of the infusion and compound conditioning stimuli. All rats began self-administration on a fixed-ratio 1 (FR1) schedule with in-session progression to a higher schedule if 7 infusions were achieved within 1-h. Schedule progression went to variable-ratio 2 (VR2), VR3, and VR5. After 3 consecutive sessions ending on VR5, rats started the next session at the next highest schedule (e.g., moving from FR1 to VR2 to start the session). The first day of self-administration was capped at 50 infusions to prevent overdose. After all self-administration sessions, animals were fed an amount to maintain 90% free-feeding body weight, and administered IV cefazolin to ensure patency. As animals progressed from an FR1 to a VR5 schedule, food restriction was gradually removed, with eventual ad libitum access to food upon reaching stability on a VR5 schedule of reinforcement. Training continued on free feeding until rats achieved stability on a VR5 schedule across three consecutive sessions within 15% coefficient of variability for infusions.

### Forced abstinence and enrichment

Animals remained in standard isolation housing (21.6 × 45.7 × 17.8 cm) or were transferred to an enriched environment for the duration of forced abstinence. EE housing consisted of 3–6 rats in a (74 × 91 × 36 cm) cage and standard bedding material. Additionally, a running wheel, tubes, toys, and nesting material was provided. Toys were exchanged every 3 days throughout forced abstinence to enhance novelty. Group assignment was counterbalanced for cocaine consumption. All rats were handled every 2 days during abstinence, including rats in isolation. The length of forced abstinence was either 1 or 21 days.

### Cue reactivity testing

After forced abstinence concluded, animals were returned to their self-administration chambers for a 1-h cue reactivity test where the previously paired lever light and tone conditioned stimuli were presented for 7-s upon a press of the active lever, but no drug was available. The timeout and house light illumination previously paired with drug infusion were present.

### Tissue collection and analysis

Immediately after the cue reactivity test, animals were anesthetized with isoflurane, decapitated, and the brain was removed and flash-frozen in methylbutane before storage in a − 70 °C freezer. Brains were dissected using a brain matrix. Based on anatomical landmarks on the ventral surface of the brain, sections 2 mm thick containing the striatum were taken with guidance from a rat brain atlas^83^. A 2.0 mm diameter punch (Harris Unicore™) of the NAcsh was then taken. RNA was isolated using standard Trizol® (#15596026; Invitrogen Corp., Carlsbad, California USA) extraction, as performed previously^38^. Samples were analyzed at the Arizona State University Genomics Core facility. RNA was prepared using the Nugen Ovation RNA-seq system (#7102-32; Tecan Genomics, Inc., Redwood City, California USA). cDNA was quantified using Nanodrop and sheared to approximately 300 base pair fragments. Libraries were generated using the Kapa Biosystem library preparation kit. Fragments were then end repaired and A-tailed. Sequencing was performed on a 1 × 75 flow cell using an Illumina NextSeq500 instrument.

### Data analysis

Fastq read files were uploaded to the public server at usegalaxy.org to analyze the data^84^. Access to the workflow can be found here: https://usegalaxy.org/u/g.powell/w/powell-et-al---environmental-enrichment-rna-seq-workflow. Quality control analysis was performed for each file using FastQC, Galaxy Version 0.69^85^ and aggregated using MultiQC, Galaxy Version 1.5.0^86^. An average of 58.43 million total reads were obtained per sample (± 3.78 million reads, SEM). Reads were aligned using HISAT2, Galaxy Version 2.1.0, a splice-aware alignment program^87^, and the Ensembl release 92 *Rattus norvegicus* reference genome^88^ with average mapping at 89.28%. Transcript assembly and quantification were performed using StringTie, Galaxy Version 1.3.3.1^89^, and an assembly reference guide acquired from Ensembl. Only alignments that matched reference transcripts were processed. All output files were then merged into a new reference transcript, keeping the original reference annotations from Ensembl, using the Stringtie merge function Galaxy Version 1.3.3 before reassembly and quantification of aligned reads with the new merged reference guide. Differential expression analysis was performed using the DESeq2 package^90–92^ in R^93^, with additional contrasts probed using the “contrasts” feature, when necessary.

### Ingenuity pathway analysis

Lists of differentially expressed transcripts with a *p* value ≤ 0.05 from DESeq2 output for each comparison were analyzed using Ingenuity Pathway Analysis (IPA, version 46901286; Qiagen, Hilden, Germany). Expression analysis settings included all available data sources using human, mouse, or rat species and the confidence level for analyses included experimentally observed outcomes and those predicted with high confidence. Measurement values for assigning directionality of expression were set to log fold change.

*RT-qPCR*. For mRNA analysis, cDNA was prepared with the SuperScript II First-Strand Synthesis system (Thermo Fisher Scientific, Waltham, Massachusetts USA). Quantification was performed using iTaq™ Universal SYBR® Green Supermix (Bio-Rad Laboratories, Hercules, California USA). Primers were designed against selected, splice variant-specific targets using Ensembl transcript sequences and the National Center for Biotechnology Information’s Primer-Blast (*Eif4e2* [ENSRNOT00000026646] Forward: GCAGAACATCCCCTGCAGTA, Reverse: GACGGACCATGTGGCTGTAA*; Camk2d* [ENSRNOT00000016026] Forward: GTTGAAGAAACCGGATGGGG, Reverse: AGCGCCGGGGTAGGAATA) and compared against the housekeeping transcript, *Gapdh* (Forward: GTGCCAGCCTCGTCTCATAG, Reverse: AAGAGAAGGCAGCCCTGGTA). Relative expression for each transcript was determined using the 2^-ΔΔCt^ method^94^, modified according to Pfaffl (2001) to incorporate real-time PCR efficiency for each gene target. With each test, a no reverse transcriptase (RT) reaction was included to act as a negative control; zero no-RT controls. Samples were run in triplicate for each transcript.

### Statistical analysis

Analysis of lever presses and cocaine infusions was performed using ANOVAs in SPSS 25 (IBM Corp., Armonk, New York USA) and Prism 8.0 (Graphpad Software, San Diego, California USA). Significance for behavioral analysis was set at 0.05. All significant results have been reported here.

## Data availability

All RNA-seq data have been deposited into the Gene Expression Omnibus with accession number GSE144606.

## Supplementary information

Supplementary figure S1

Supplementary figure S2

Supplementary figure S3

Supplementary figure S4

Supplementary figure S5

Supplementary figure S6

Supplementary table S1

Supplementary table S2

Supplementary table S3

Supplementary table S4

Supplementary Legends
