# Supplementary figures and images for: Environmental enrichment during forced abstinence from cocaine self-administration opposes gene network expression changes associated with the incubation effect
Source: Sci Rep. 2020 Jul 9;10:11291. doi: 10.1038/s41598-020-67966-8 (PMC7347882; doi:10.1038/s41598-020-67966-8)

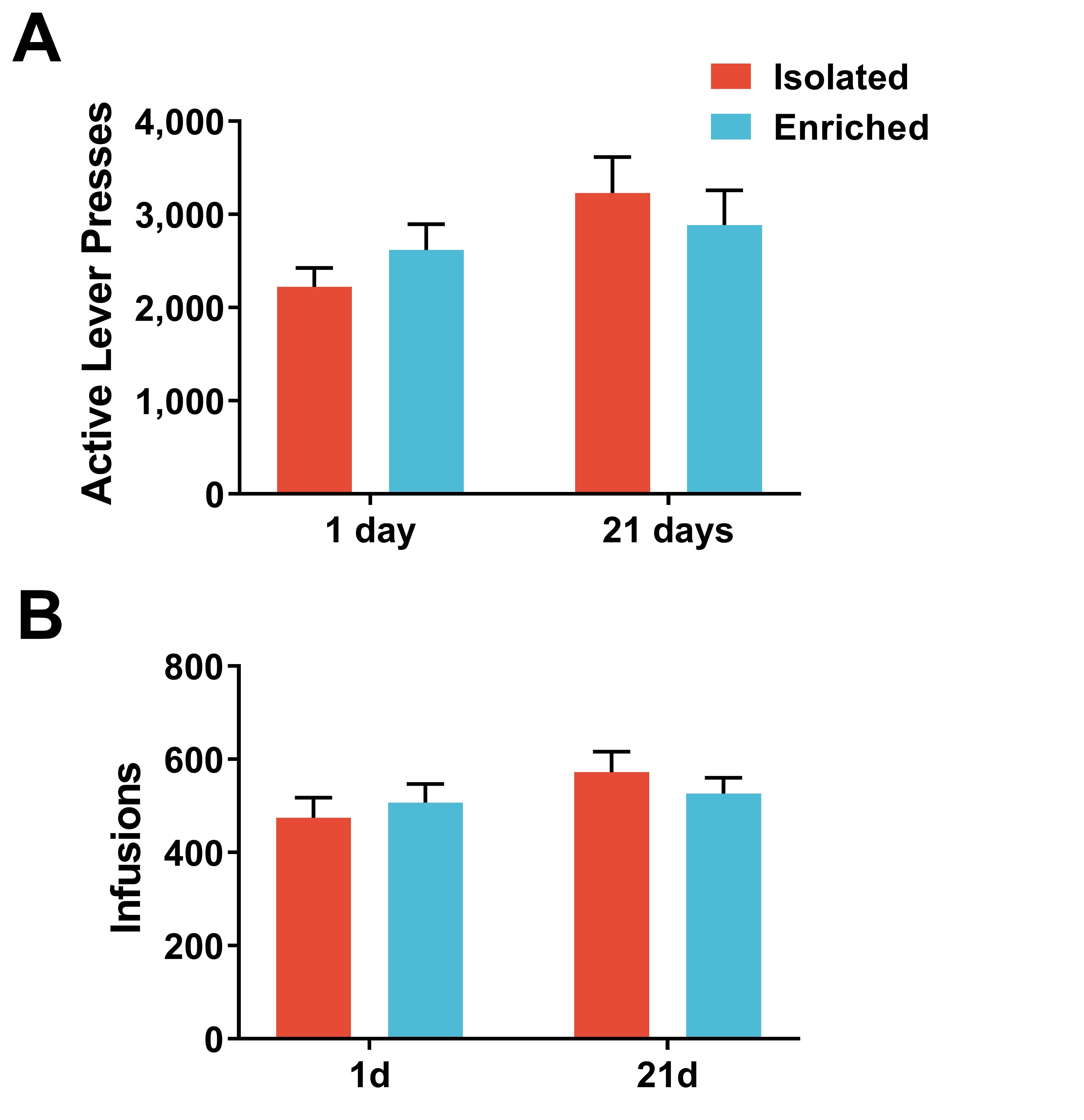

Supplement: Supplementary file 1 — Supplementary figure S1 [file 41598_2020_67966_MOESM1_ESM.jpg]

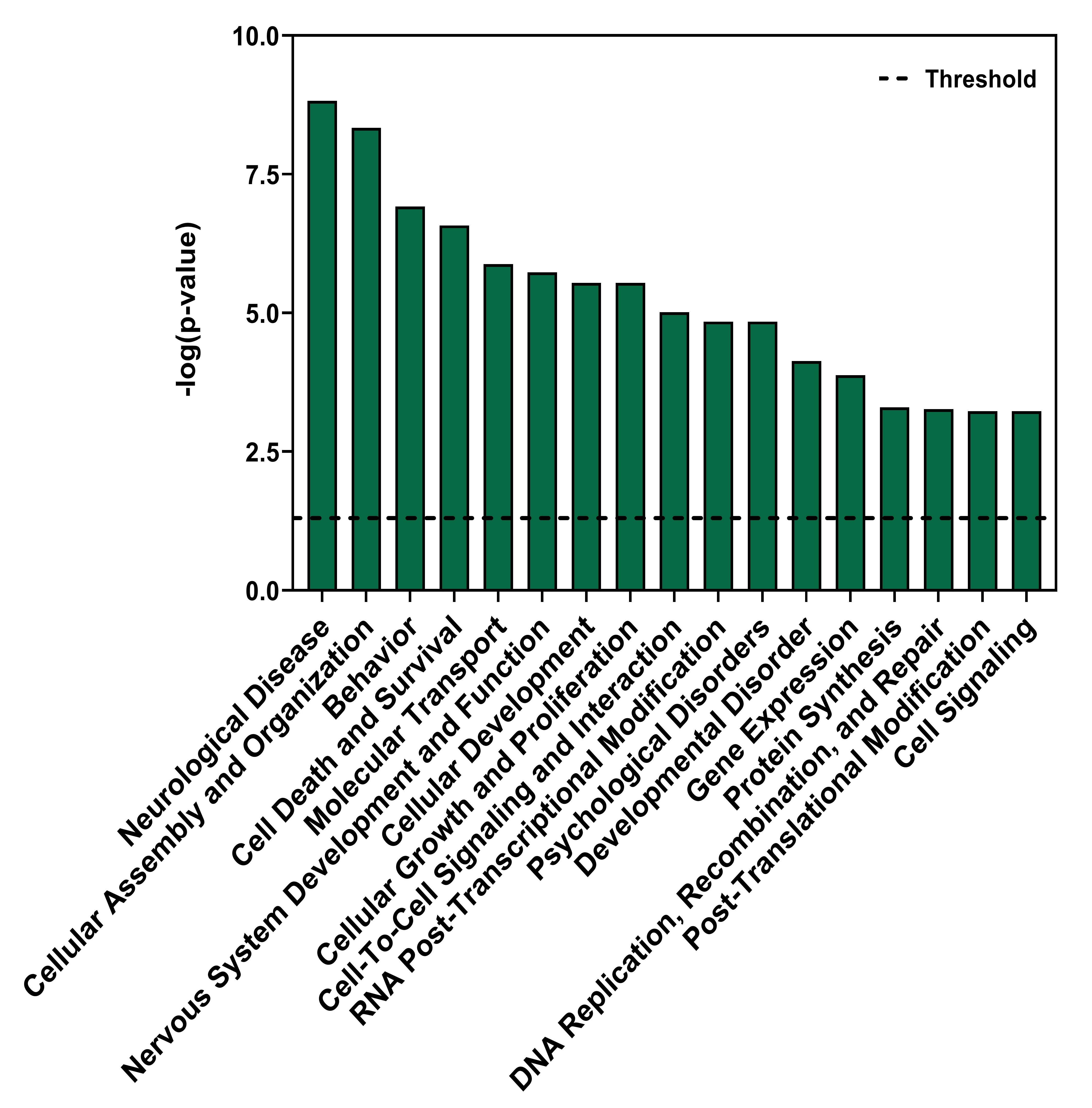

Supplement: Supplementary file 2 — Supplementary figure S2 [file 41598_2020_67966_MOESM2_ESM.jpg]

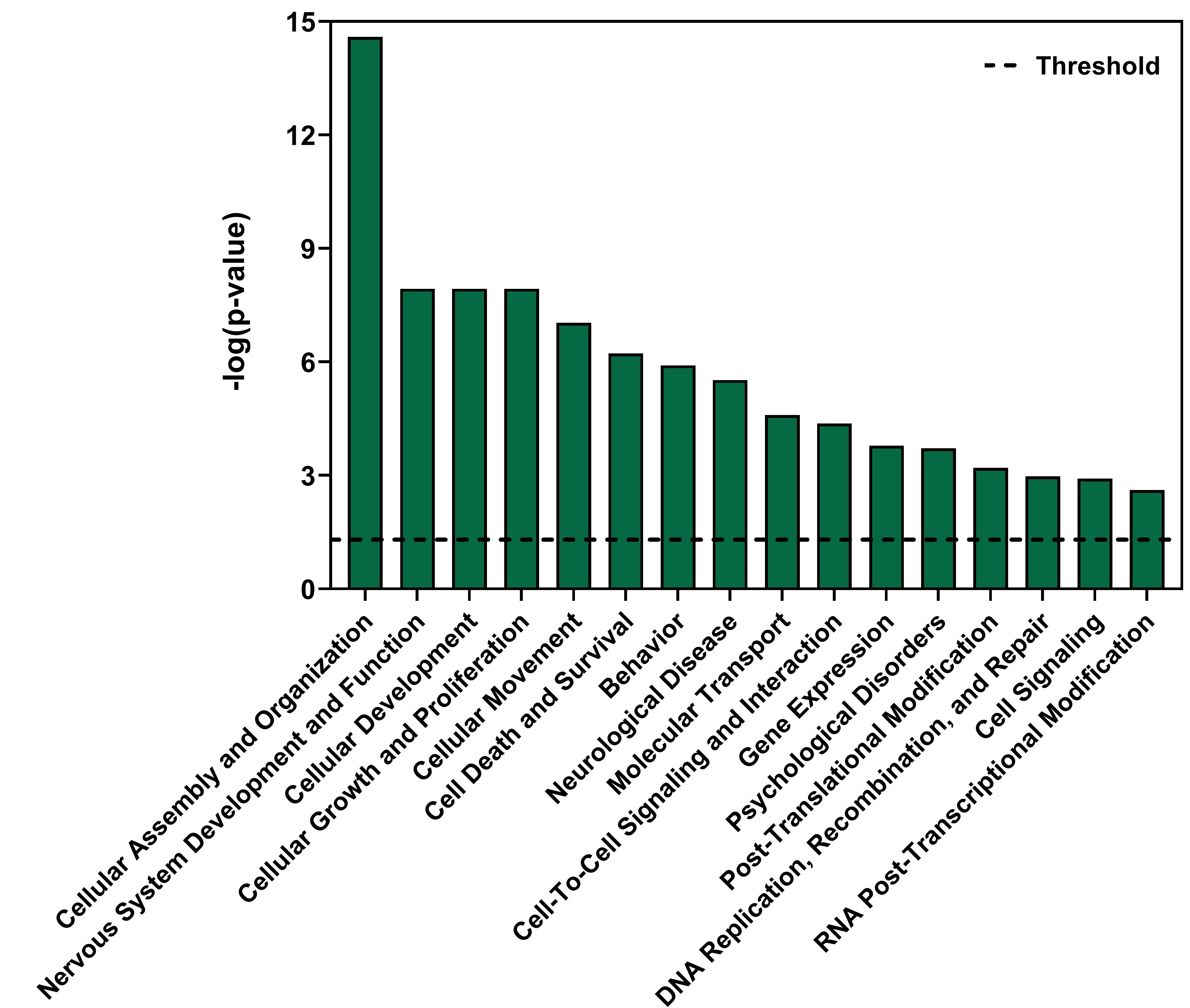

Supplement: Supplementary file 3 — Supplementary figure S3 [file 41598_2020_67966_MOESM3_ESM.jpg]

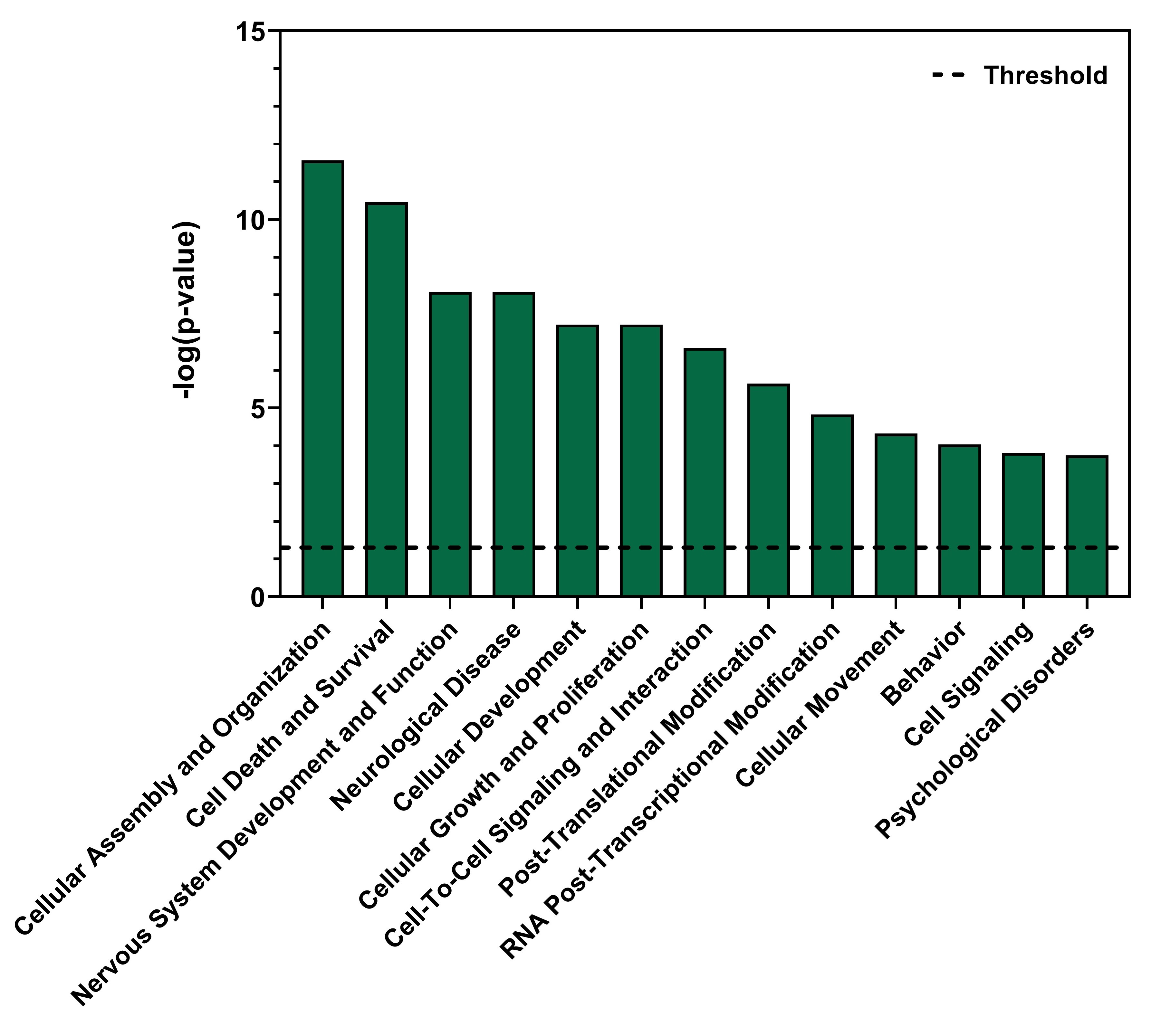

Supplement: Supplementary file 4 — Supplementary figure S4 [file 41598_2020_67966_MOESM4_ESM.jpg]

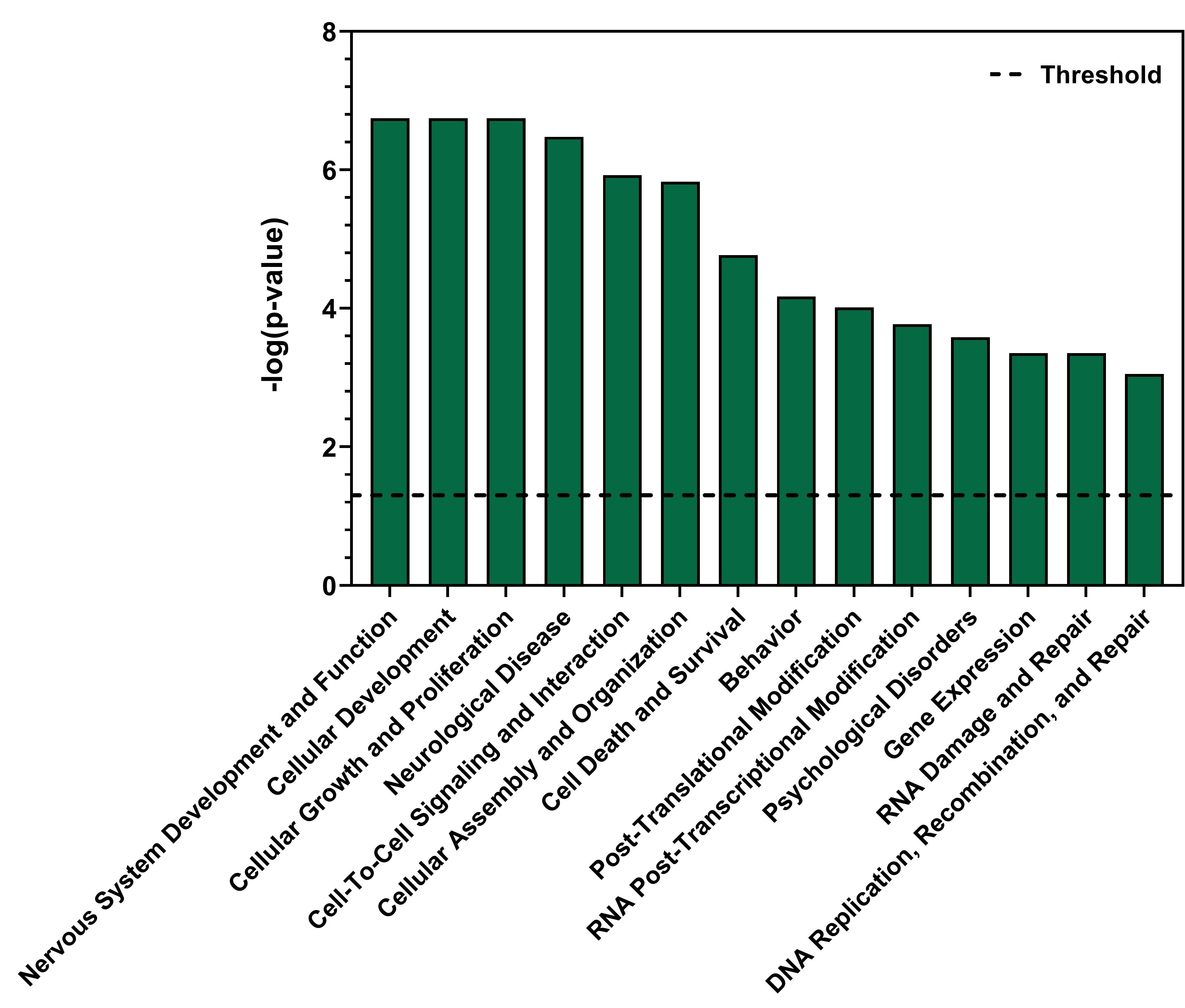

Supplement: Supplementary file 5 — Supplementary figure S5 [file 41598_2020_67966_MOESM5_ESM.jpg]

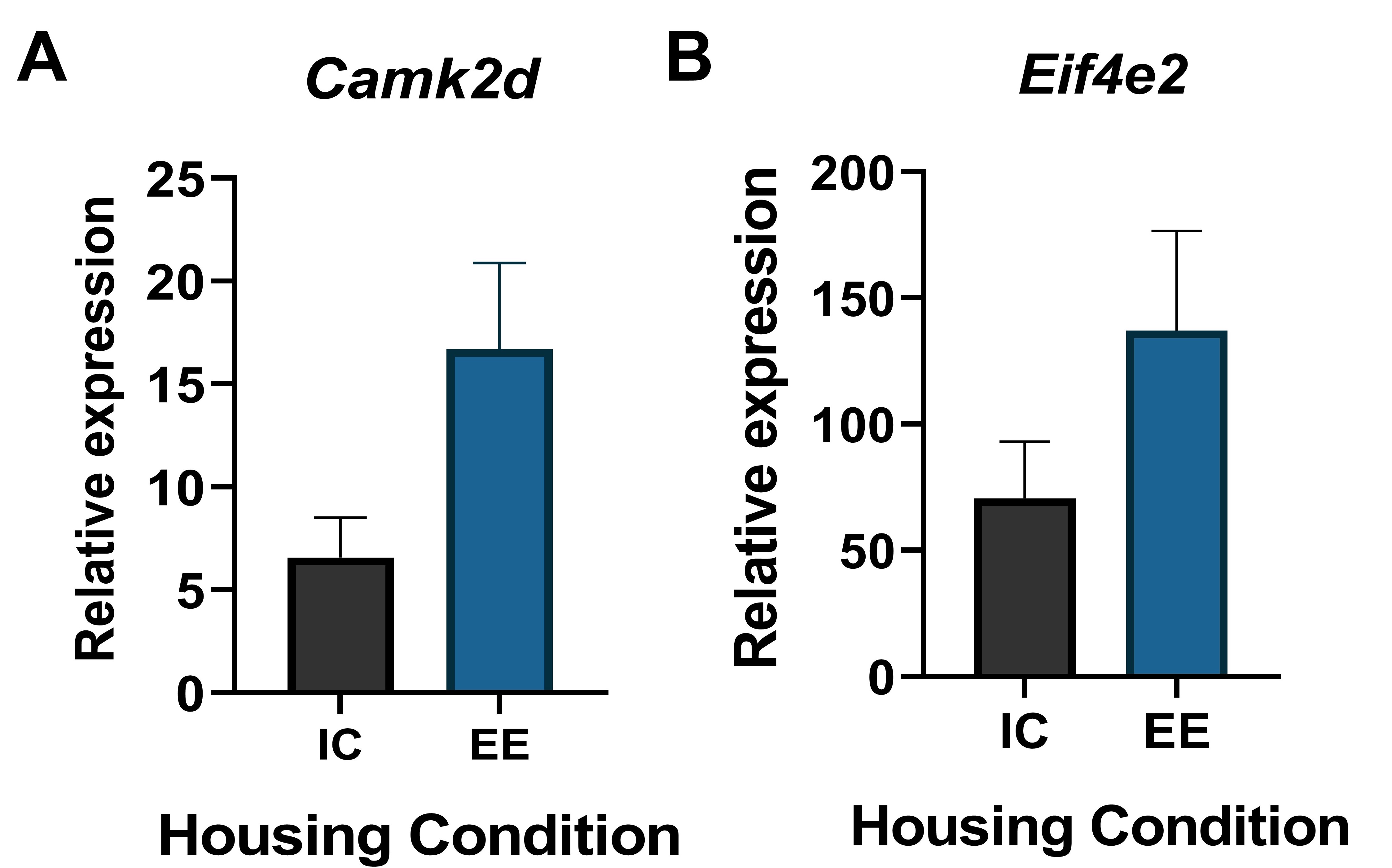

Supplement: Supplementary file 6 — Supplementary figure S6 [file 41598_2020_67966_MOESM6_ESM.jpg]
