# Supplementary material for: Environmental enrichment during forced abstinence from cocaine self-administration opposes gene network expression changes associated with the incubation effect
Source: Sci Rep. 2020 Jul 9;10:11291. doi: 10.1038/s41598-020-67966-8 (PMC7347882; doi:10.1038/s41598-020-67966-8)
Supplement: Supplementary file 11 — Supplementary Legends [file 41598_2020_67966_MOESM11_ESM.docx]

**Supplementary Materials**

**Title:** Environmental enrichment during forced abstinence from cocaine self-administration opposes gene network expression changes associated with the incubation effect

**Authors**: Gregory L. Powell^1^, Annika Vannan^1^, Ryan M. Bastle^1,4^, Melissa A. Wilson^1,2^, Michela Dell’Orco^3^, Nora I. Perrone-Bizzozero^3†^, Janet L. Neisewander^1*†^

**Affiliations**: ^1^School of Life Sciences, Arizona State University, Tempe, AZ, USA; ^2^Center for Evolution and Medicine, Arizona State University, Tempe, AZ, USA; ^3^Department of Neurosciences, University of New Mexico School of Medicine, Albuquerque, NM, USA; ^4^Current address Nash Family Department of Neuroscience, Icahn School of Medicine at Mount Sinai, New York, NY, USA

^†^Equal senior author role

*Correspondence may be sent to:

Janet L. Neisewander, Ph.D.

School of Life Sciences

Arizona State University

PO Box 874501

Tempe, AZ 85287-4501

Figure S1. Similarity of the groups during acquisition and maintenance of cocaine self-administration. (A) Total number of active lever presses (+SEM) and (B) total number of infusions earned during self-administration in isolated (IC) and enriched (EE) rats prior to 1 or 21 days (d) of forced abstinence. No differences were observed across groups later assigned to housing environment and abstinence length conditions.

Figure S2. Selection of top diseases and functions impacted by abstinence. Threshold: pathways with a –log(P)>1.301 were considered significantly regulated.

Figure S3. A selection of top diseases and functions for the housing effect. Threshold: pathways with a –log(P)>1.301 were considered significantly regulated.

Figure S4. A selection of top diseases and functions for the incubation effect. Threshold: pathways with a –log(P)>1.301 were considered significantly regulated.

Figure S5. A selection of top diseases and functions for the enrichment effect. Threshold: pathways with a –log(P)>1.301 were considered significantly regulated.

Figure S6. RT-qPCR validation. (A) Relative expression of *Camk2d* compared to *Gapdh* in isolated (IC) and enriched (EE) rats following 21 days of forced abstinence from self-administration. (B) Relative expression of *Eif4e2* compared to *Gapdh* in IC and EE rats.

Table S1. IPA output for differentially expressed mRNAs related to the Forced Abstinence effect, including Canonical Pathways, Diseases and Functions, and Upstream Analysis.

Table S2. IPA output for differentially expressed mRNAs related to the Housing effect, including Canonical Pathways, Diseases and Functions, and Upstream Analysis.

Table S3. IPA output for differentially expressed mRNAs related to the Incubation effect, including Canonical Pathways, Diseases and Functions, and Upstream Analysis.

Table S4. IPA output for differentially expressed mRNAs related to the Enrichment effect, including Canonical Pathways, Diseases and Functions, and Upstream Analysis.
